# Bovine delta papillomavirus E5 oncoprotein negatively regulates the cGAS-STING signaling pathway in cattle in a spontaneous model of viral disease

**DOI:** 10.3389/fimmu.2022.937736

**Published:** 2022-10-12

**Authors:** Francesca De Falco, Anna Cutarelli, Adriana Florinela Catoi, Barbara Degli Uberti, Bianca Cuccaro, Sante Roperto

**Affiliations:** ^1^ Dipartimento di Medicina Veterinaria e Produzioni Animali, Università degli Studi di Napoli Federico II, Napoli, Italy; ^2^ Istituto Zooprofilattico Sperimentale del Mezzogiorno, Portici, Italy; ^3^ Physiopathology Department, Faculty of Medicine “Iuliu Hatieganu”, University of Medicine and Pharmacy, Cluj-Napoca, Romania

**Keywords:** bovine papillomavirus E5 oncoprotein, cGAS, stimulator of interferon genes (STING), IKK kinase complex, transcription factor IRFs, ELKS, TAK1

## Abstract

Persistent infection and tumorigenesis by papillomaviruses (PVs) require viral manipulation of various cellular processes, including those involved in innate immune responses. The cyclic guanosine monophosphate-adenosine monophosphate synthase-stimulator of interferon genes (cGAS-STING) pathway has emerged as an essential innate immune sensing system, that recognizes DNA and trigger potent antiviral effector responses. In this study, we found that bovine PV (BPV) E5 protein, the major oncoprotein of bovine delta PVs, interacts with STING but not with cGAS in a spontaneous BPV infection of neoplastic urothelial cells of cattle. Real-time RT-PCR revealed a significant reduction in both cGAS and STING transcripts in E5-expressing cells. Furthermore, western blot (WB) analysis failed to detect any variation in the expression of interferon-inducible protein 16 (IFI16), an upstream effector of the STING pathway. A ternary complex composed of E5/STING/IFI16 was also observed. Co-immunoprecipitation studies showed that STING interacts with a protein network composed of total and phosphorylated TANK-binding kinase 1 (TBK1), total and phosphorylated interferon regulatory factor 3 (IRF3), IRF7, IKKα, IKKβ, IKKϵ, ELKS, MEKK3, and TAK1. RT-qPCR revealed a significant reduction in TBK1 mRNA levels in BPV-infected cells. WB analysis revealed significantly reduced expression levels of pTBK1, which is essential for the activation and phosphorylation of IRF3, a prerequisite for the latter to enter the nucleus to activate type 1 IFN genes. WB also revealed significantly down-expression of IKKα, IKKβ, IKKϵ, and overexpression of IRF7, ELKS, MEKK3, and TAK1in BPV-positive urothelial cells compared with that in uninfected healthy cells. Phosphorylated p65 (p-p65) was significantly reduced in both the nuclear and cytosolic compartments of BPV-infected cells compared with that in uninfected urothelial cells. Our results suggest that the innate immune signaling pathway mediated by cGAS-STING is impaired in cells infected with BPV. Therefore, effective immune responses are not elicited against these viruses, which facilitates persistent viral infection and subsequent tumorigenesis.

## Introduction

The cyclic guanosine monophosphate-adenosine monophosphate synthase-stimulator of interferon genes (cGAS-STING) pathway has emerged as a central innate immune sensing pathway that recognizes pathological DNA and triggers potent antiviral effector responses (1, 2). The cGAS-STING pathway not only mediates protective immune defence against infections by a large variety of DNA-containing pathogens but also detects tumor-derived DNA, thus playing a pivotal role in antitumor immunity (3, 4). Double-stranded DNA (dsDNA) binds to and activates cGAS, which converts guanosine 5′-triphosphate (GTP) and adenosine 5′-triphosphate (ATP) to cGAMP; this molecule is a second messenger that binds to and activates STING, an endoplasmic reticulum (ER) adaptor protein, that mediates innate immune activation (5, 6). STING activation requires its translocation from the ER to the Golgi apparatus and subsequent polymerization, which is supposedly mediated by sulphated glycosaminoglycans (sGAG) (7). In the trans-Golgi network, STING recruits the IKK kinase complex and TANK-binding kinase 1 (TBK1), which, in turn, phosphorylate STING and the transcription factor IRF3 (8). This triggers a signaling cascade that leads to the production of immune and inflammatory mediators, including type I and type III interferons, and provides a robust antiviral response (4). However, the mechanism of activation of TBK1 and IRF3 by cGAMP-bound STING is not yet understood (8).

Although the cGAS-STING-mediated DNA-sensing signal is crucial for host defence against many viruses, especially DNA viruses, few viral components have been identified that specifically target this signaling pathway (9). Many human oncogenic viruses such as human papillomavirus (HPV) (1, 10–13), many DNA viruses such as herpesvirus, pseudorabies virus (14), avian oncogenic herpesvirus (15), poxviruses such as fowlpox virus (16), and asfarviruses such as African swine fever virus (17–20) are known to be recognized by host pattern-recognition receptors and have been shown to subvert the cGAS-STING signaling pathway.

*Delta* papillomavirus (δPV) is one of the five genera of bovine papillomaviruses (BPVs). Currently, this genus comprises four BPVs, namely BPV-1, BPV-2, BPV-13, and BPV-14, which are believed to be highly pathogenic and frequently associated with cutaneous and mucosal tumors in cattle (21). BPVs are typically responsible for latent infections. BPV DNA has been found to be highly prevalent in healthy cattle (22), as well as in small ruminants that were free at pasture (23, 24). Establishment of a persistent BPV infection is a major risk factor for cancer development and requires the virus to evade the first line of defence the innate immune system. BPV-2 and BPV-13 are the most important infectious agents that cause bladder cancer in some breeds of pasture-residing cattle that graze on lands with an overgrowth of bracken ferns (*Pteridium aquilinum*). BPV-2 and BPV-13 cause both abortive and productive infections, resulting in neoplastic and non-neoplastic pathologies. These viruses exert oncogenic properties prevalently *via* the E5 oncoprotein, which is believed to be the major oncoprotein of δPVs. It is a frequently studied protein that is small in size (40–85 amino acids) and displays transforming activity *via* numerous pathways, even in the absence of other viral genes.

The co-evolution of oncogenic BPVs with transforming urothelial cells suggests that BPVs can develop mechanisms that lead to the shutdown of the host immune system, which allows BPVs to escape immune responses. Recently, BPV E5 oncoprotein was shown to negatively regulate the host antiviral innate immune response *via* retinoic acid-inducible gene I (RIG-I)-like receptors (RLRs) in a spontaneous model of bovine papillomavirus disease in cattle (25).

We aimed to investigate the effect of the BPV E5 oncoprotein on the cGAS-STING signaling pathway, which results in impaired interferon and NF-κB responses, in a spontaneous model of papillomavirus disease in cattle.

## Material and methods

### Animal samples

The bladder samples of this study were previously analyzed for Rig-I-like receptors. Briefly, bladder mucosa samples from 15 cows clinically suffering from chronic enzootic hematuria were collected from public slaughterhouses after bladder neoplasms had been identified during mandatory post-mortem examination. These animals were categorized as “infected” as they harbored papillomavirus infection. Further bladder mucosa samples from 15 apparently healthy cows were also collected. Six of these apparently healthy bladders showed a microscopic pattern characterized by inflammatory cells composed of small foci of lymphocytes beneath the urothelium. These animals were categorized as “non-infected” as they did not harbor any papillomavirus infection. The remaining nine animals were categorized as “healthy” since neither inflammatory cells nor papillomavirus infection were seen in their bladder samples. Animals from these groups were 3-5 years old. All bladder samples were immediately subdivided and either fixed in 10% buffered formalin for microscopic investigation or frozen in liquid nitrogen and stored at –80°C for subsequent molecular biology analysis.

### Antibodies

Rabbit antibodies against cGAS, IFI16, STING, total and phospho-TBK1 (pTBK1), total and phospho-IRF3 (pIRF3), IRF7, TAK1, MEKK3, total and phospho-p65, p52, RelB, IKKα, IKKβ, IKKε and anti-mouse IKK γ, were obtained from Cell Signaling Technology (Leiden, Netherlands). Mouse anti-ELKS, anti-cMyc, and β-actin antibodies were purchased from Santa Cruz Biotechnology (TX, USA). Rabbit polyclonal anti-E5 serum recognising the C-terminal 14 amino acids of the BPV E5 oncoprotein was a kind gift provided by Prof. DiMaio (Yale University, New Haven, USA).

### RNA extraction and reverse transcription (RT)-PCR

Total RNA was extracted from bladder samples from 15 cows suffering from chronic enzootic hematuria, 9 healthy and 6 “non-infected” cows using the RNeasy Mini Kit (Qiagen, NW, DE), according to the manufacturer’s instructions. Genomic DNA was removed from the RNA preparations using RNase-free DNase Fermentas Life Sciences (Thermo Fisher Scientific, MA, USA). A total of 1 μg RNA was used to generate single-stranded cDNA, using the QuantiTect Reverse Transcription Kit (Qiagen NW, DE), according to the manufacturer’s instructions. PCR was performed with a specific primer set designed using Primer3, an online tool, for bovine cGAS, STING, TBK1, IRF3, IRF7, IKK ε genes. The following primers were used: cGAS forward: 5’-AGGAGATATCCGTAGCGGC-3’: reverse 5’-TCATTAGGAGCAGAAATCTTCACT-3’; STING forward: 5’-GTGCAGTGTGTATGCTTGGC-3’: reverse 5’-GGCTGGAGTGAGGCATCTTC-3’; TBK1 forward: 5’- CACCAAGCTGTTGAGACTTTCC-3’: reverse 5’-AGTGCCTTCTTGATGGGTCC-3’, IRF3 forward: 5’- TTTGTGAACTCAGGAGTCAGGG -3’: reverse 5’-CCTGGGCTCAAGTCCATGTC-3, IRF7 forward: 5’-CCGCCGATCCCTGAGAGTG-3’: reverse: 5’-AAGCCTAGGCCTTCTGGGC-3’: IKKε forward: 5’-CGCTTCCGGACCAGTATCAA-3’: reverse: 5’-CCTCCAGGGAAACCCATAGC-3’. Conditions for PCR were as follows: 94°C for 5 min, 40 cycles at 95°C for 30 s, 58°C for 30 s, and 72°C for 30s.

### One-step reverse transcription (RT)-ddPCR

Total RNA was extracted from 6 bladders categorized as ‘non-infected’ and 9 bladders as ‘healthy’. 100 ng of total RNA was used for One-Step RT-ddPCR Advanced Kit for Probes (Bio-Rad Laboratories, Hercules, CA, USA) according to the manufacturer’s instructions. The reaction was performed in a final volume of 22 μL, containing 11 μL of ddPCR Supermix 2x for Probes, 2 μL reverse transcriptase, 1 μL DTT and 1 μL of primer and probe mix for all 4 delta BPVs. The sequences of primers and probes used to verify the presence of the delta BPV transcripts were previously used (22–24). The plate was transferred to an automated droplet generator (AutoDG, Bio-Rad Laboratories, Hercules, CA, USA). PCR amplification was carried out on a T100 Thermal Cycler (Bio-Rad Laboratories Hercules, CA, USA) with the following thermal profile: 50°C for 60min, 95°C for 10 min, 40 cycles of 94°C for 30 s, 58°C for 1 min, 1 cycle at 98°C for 10 min, and ending at 4°C. After amplification, the plate was loaded onto a droplet reader (Bio-Rad Laboratories, Hercules, CA, USA), and the droplets from each well of the plate were read automatically. Each sample was analyzed in triplicate.

### Sequence analysis

PCR products, obtained by RT-PCR, were purified using the QIAquick PCR Purification Kit (Qiagen NW, DE) and were subjected to bidirectional sequencing using the Big Dye-Terminator v1.1 Cycle Sequencing Kit (Applied Biosystems, CA, USA), according to the manufacturer’s recommendations. Dye terminators from sequences were removed using a DyeEx-2.0 Spin Kit (Qiagen), and sequences were run on a SeqStudio Genetic Analyzer (Thermo Fischer Scientific, CA, USA). Electropherograms were analyzed using Sequencing Analysis v5.2 and Sequence Scanner v1.0 software (Thermo Fischer Scientific, CA, USA). The sequences were analyzed using the BLAST program.

### Real-time RT- PCR

To perform real-time RT-PCR analysis, total RNA and cDNA from diseased and healthy urinary bladder samples were generated, as described above. Real-time PCR was performed with a Bio-Rad CFX Connect™ Real-Time PCR Detection System (Bio-Rad, Hercules, CA, USA), using iTAq Universal SYBR^®^ Green Supermix (Bio-Rad). Each reaction was performed in triplicate, and the primers used for cGAS, STING, TBK1, IRF3, IRF7, and IKK**ϵ** were the same as those used for RT-PCR. The PCR thermal profile was as follows: 95°C for 10 min, 40 cycles of 94°C for 15 s, and 58°C for 30 s, followed by a melting curve. Relative quantification (RQ) was calculated using the CFX Manager™ software, based on the equation RQ=2^−ΔΔCq^, where Cq is the quantification cycle to detect fluorescence. Cq data were normalized to the bovine β-actin gene (forward: 5′- TAGCACAGGCCTCTCGCCTTCGT-3′, reverse 5′-GCACATGCCGGAGCCGTTGT-3′).

### Western blot (WB) analysis

Healthy, non-infected and infected bovine urothelial samples were lysed in radioimmunoprecipitation assay (RIPA) buffer (50 mM Tris- HCl [pH 7.5], 1% Triton X-100, 400 mM NaCl, 1 mM ethylenediaminetetraacetic acid, 2 mM phenylmethylsulfonyl fluoride, 1.7 mg/mL aprotinin, 50 mM NaF, and 1 mM sodium orthovanadate). Protein concentration was measured using the Bradford assay (Bio-Rad). For western blotting, 50 μg protein lysate was heated at 90°C in 4X premixed Laemmli sample buffer (Bio-Rad), clarified by centrifugation, separated by sodium dodecyl sulphate–polyacrylamide gel electrophoresis, and transferred onto nitrocellulose membranes (GE Healthcare, UK). Membranes were blocked with EveryBlot Blocking Buffer (Bio-Rad, Hercules, CA, USA) for 30 min at room temperature and subsequently incubated overnight at 4°C with primary antibodies diluted in EveryBlot Blocking Buffer. The membranes were washed three times with TBST, incubated for 1 h at room temperature with goat anti-rabbit or goat anti-mouse (Bio-Rad, Hercules, CA, USA) HRP- conjugated secondary antibody, diluted at 1:5,000 in TBST containing EveryBlot Blocking Buffer, and washed three times with TBST. Immunoreactive bands were detected using Western Clarity Western ECL Substate or Clarity Max Western ECL Substate (Bio-Rad, Hercules, CA, USA) and ChemiDoc XRS Plus (Bio-Rad, Hercules, CA, USA). Images were acquired using Image Lab Software, version 6.1.

### Nuclear and cytosolic fractioning

Subcellular fractioning was obtained from bladder samples of 15 infected cows, 9 healthy and 6 non-infected tissues. 30 mg of frozen tissues were cut into ~2mm^2^ pieces, transferred on 2 mL reaction tube and added to 400 μL of cytosolic extraction buffer (30 mM Hepes-KOH pH 7.6, 20 mM KCl, 10 mM MgCl2, 0.2 mM EDTA pH 8.0, 20% Glycerol). The samples were homogenized using the TissueLyser LT (Qiagen NW, DE) for 10 min and centrifuged at 600g for 1 min to 4°C. About 150–200 μL of supernatant was centrifuged and the obtained supernatant was carefully recovered and clarified with a second centrifugation step at 14000g for 10min to 4°C, obtaining thus the cytoplasmic fraction. To ensure that tissues were completely lysed, two washing steps are performed on pellet with increased sucrose concentration, using 150 μL of WASH150 (cytosolic extraction buffer 1X with 150 mM sucrose) and then 150 μL of WASH250 (cytosolic extraction buffer 1X with 250 mM sucrose). Finally, 150 μL for tissues extracts of NEB buffer **(**350 mM sucrose, 15 mM Hepes-KOH pH 7.6, 385 mM KCl, 5 mM MgCl2, 0.1 mM EDTA pH 8.0, 0.05% Tween 20, 10% Glycerol) was used to re-suspend the pellet. The high sucrose concentration and detergent in NEB buffer ensure the lysis of nuclear membrane. Vigorous vortexing was performed to ensure a complete solubilisation of the pellet. A centrifugation step to 14000g for 10min to 4°C was performed to recover the soluble protein fraction from lysed nuclei thus obtaining the nuclear fraction.

### Immunoprecipitation

Total protein extracts from infected, non-infected, and healthy bladder samples, obtained as previously described, were immunoprecipitated. Protein samples (1mg) were incubated with anti-cGAS or anti-STING or antirabbit IgG (isotype), anti-IFI16 or anti-rabbit IgG (isotype) (Bethyl Laboratories, Inc., TX, USA) for 1 h at 4°C with gentle shaking. Thereafter, the samples were centrifuged at 1,000 g for 5min at 4°C and incubated with 20 μL of Protein A/G-Plus Agarose (sc-184 2003) (Santa Cruz Biotechnology) overnight at 4°C. The immunoprecipitates were washed four times in complete lysis buffer and separated on polyacrylamide gels. Subsequently, the proteins were transferred onto nitrocellulose membranes. Membranes were blocked with EveryBlot Blocking Buffer (Bio-Rad, Hercules, CA, USA) for 30 min at room temperature. The membranes were subsequently incubated overnight at 4°C with primary antibodies diluted in EveryBlot Blocking Buffer, washed three times with TBST, incubated for 1 h at room temperature with goat anti-rabbit or goat anti-mouse (Bio-Rad, Hercules, CA, USA) HRP- conjugated secondary antibody, diluted at 1:5,000 in TBST containing EveryBlot Blocking Buffer, and washed three times with TBST. Immunoreactive bands were detected using Clarity Western ECL Substate or Clarity Max Western ECL Substate (Bio-Rad, Hercules, CA, USA) and ChemiDoc XRS Plus (Bio-Rad, Hercules, CA, USA). Images were acquired using Image Lab Software, version 6.1.

### Statistical analysis

Results are presented as the mean ± standard error (SE). Data were assessed by one-way analysis of variance (ANOVA), followed by Tukey’s test for multiple comparisons of means using the GraphPad PRISM software version 9 (GraphPad Software, San Diego, CA, USA). A p-value ≤ 0.05 indicated statistical significance.

## Results

### Virological findings

BPV-2 and BPV-13 are considered the most important infectious agents associated with urothelial tumors in cattle (26–29). The E5 oncoprotein is a major protein that displays transforming activity in both mesenchymal and epithelial cells to form benign and malignant tumors (30). Detailed virological findings involving these samples have been reported previously. Briefly, we detected E5 oncoprotein transcripts by RT-PCR, the sequencing of which showed 100% identity with BPV-2 and BPV-13 in 15 neoplastic bladders of cattle (Accession numbers M20219.1 and JQ798171.1, respectively) (25). One-Step RT-ddPCR analysis failed to detect any E5 mRNA from four bovine *Delta* papillomaviruses in both “non-infected” and healthy bladders (**Supplemental Figures 1A, B**), thus showing that these animals did not harbor any abortive papillomavirus infection.

### cGAS-STING pathway

**Figure 1:**
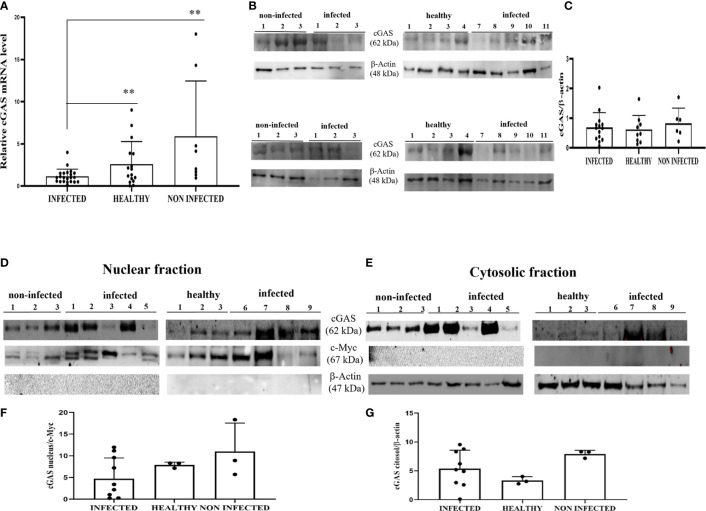
**(A)** RT-qPCR analysis of cGAS mRNA levels in infected, non-infected, and healthy bladder samples, respectively. cGAS mRNA was significantly reduced in infected bladder samples compared with that in both non-infected and healthy bladder samples (**p ≤ 0.01). **(B)** Western blot analysis of cGAS in infected, non-infected, and healthy bladder samples. **(C)** Densitometric analysis was performed by comparing the protein expression levels of total cGAS with β-actin. **(D, E)** cGAS western blot of nuclear and cytosolic fractions of infected, non-infected, and healthy bladder tissues. **(F, G)** cMyc and β-actin was used to verify the selectivity of the extracts and perform the densitometric analysis. There was no statistically significant variation in cGAS protein levels in total and in differentiated fractions in infected bladder tissue compared with both non-infected and healthy bladder samples. Plots present measurements from each sample performed in triplicates.

**Figure 2:**
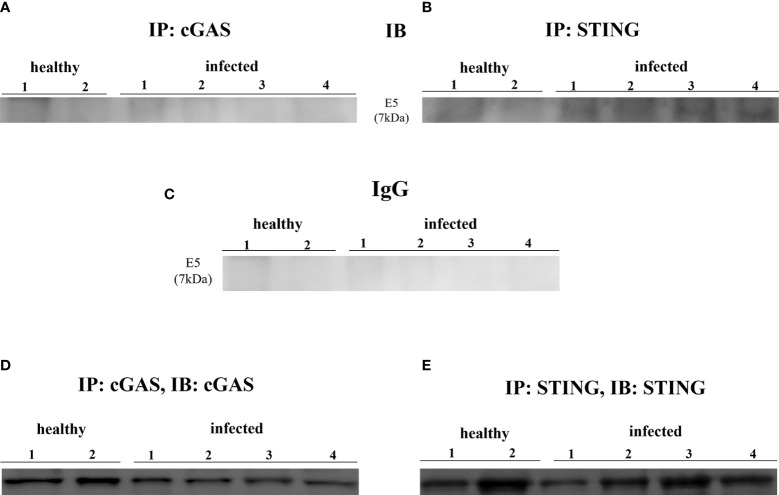
**(A, B)** cGAS and STING immunoprecipitation assay. Only STING bound E5 oncoprotein. **(C)** Rabbit IgG immunoprecipitation control. **(D, E)** Immunoprecipitation input.

Experimental studies have shown that HPV E6 and E7 oncogenes hamper the cGAS-STING signaling pathway as they are able to reduce cGAS mRNA expression (11, 12, 31). We investigated the involvement of cGAS-STING axis in a spontaneous model of persistent BPV infection of urothelial cells resulting in bladder tumors in cattle. To test whether cGAS mRNA is reduced in spontaneous BPV-infected cells, we carried out RT-PCR using specific primers for bovine cGAS. The sequencing of RT-PCR-generated transcript amplicons (**Supplemental Figure 2A**) revealed cDNA fragments showing 100% identity with *Bos taurus* cGAS mRNA sequence deposited in GenBank (Accession number: XM_002690020.6) (**Supplemental Figure 2B**). Real time RT-PCR revealed a significant reduction in cGAS transcripts in BPV-infected cells compared with that in non-infected and healthy cells (**p ≤ 0.01) (**Figure 1A**). We then investigated the expression levels of cGAS protein by WB analysis. No significant differences in cGAS protein expression levels were observed among infected, non-infected, and healthy urothelial cells (**Figures 1B, C**). Since cGAS possibly plays a crucial role in activating cellular innate immune responses upon nuclear engagement due to viral infection (2, 32), we investigated the expression levels of cGAS protein in the nuclear and cytosolic compartments. No significant differences were observed in the nuclear or cytosolic cGAS among infected, non-infected, and healthy urothelial cells, even though a slight reduction in protein levels was observed in the nuclear fraction of neoplastic cells (**Figures 1D–G**). To evaluate whether cGAS interacts with the E5 oncoprotein, we performed co-immunoprecipitation studies using anti-cGAS antibodies. We did not detect any interactions involving the viral protein (**Figure 2A**).

### Stimulator of interferon genes (STING)

**Figure 3:**
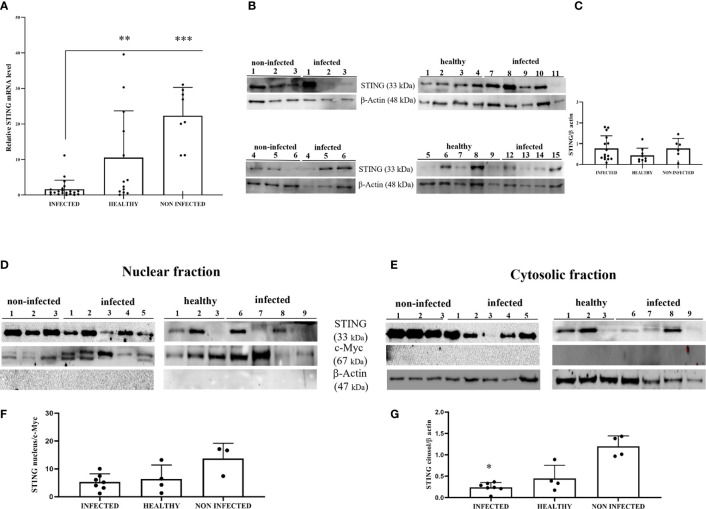
**(A)** RT-qPCR analysis of STING mRNA levels in infected, non-infected, and healthy bladder samples, respectively. STING mRNA expression was significantly reduced in infected bladder compared with both non-infected and healthy bladder samples (***p ≤ 0.001; **p ≤ 0.01);. **(B)** Western blot analysis of STING in infected, non-infected, and healthy bladder samples. **(C)** Densitometric analysis was performed by comparing the protein expression levels of total STING with β-actin. **(D, E)** STING western blot of nuclear and cytosolic fractions of infected, non-infected, and healthy bladder tissues. **(F, G)** cMyc and β-actin were used to verify selectivity of the extracts and perform the densitometric analysis. There was no statistically significant variation in STING protein levels in total and in differentiated fractions in infected bladder tissue compared with both non-infected and healthy bladder samples. Plots present measurements from each sample performed in triplicates.

STING is an ER-associated transmembrane protein (33) that is activated by cGAMP and interferon-inducible protein 16 (IFI16) for IFN-1 and NF-κB induction (2, 34). Experimental studies have shown that HPV18 oncoproteins are responsible for transcriptional reduction of STING expression (31). We performed RT-PCR using specific primers for bovine STING. The sequencing of RT-PCR-generated transcript amplicons (**Supplemental Figure 2A**) revealed cDNA fragments showing 100% identity with *Bos taurus* STING mRNA sequence deposited in GenBank (Accession number: NM_001046357-2) (**Supplemental Figure 2C**). RT-qPCR revealed a significant reduction in STING transcripts in BPV-positive urothelial cells compared with that in BPV-negative, non-infected, and healthy cells (***p ≤ 0.001; **p ≤ 0.01, respectively) (**Figure 3A**). To investigate the expression levels of STING protein, we performed WB analysis on total protein extracts. WB analysis did not reveal any statistical differences in STING expression levels among infected, non-infected, and healthy urothelial cells (**Figures 3B, C**). Since STING localizes in both the nucleus and cytoplasm of cells (35), the relative distribution of STING between these compartments was investigated by WB. Nuclear distribution of STING did not vary significantly among infected, non-infected, and healthy urothelial cells, although a slight reduction in its expression levels was observed in the nucleus of neoplastic urothelial cells (**Figures 3D, F**). STING expression levels were significantly reduced in the cytosolic compartment of BPV-infected urothelial cells compared with that of BPV-negative uninfected cells (**p ≤ 0.05) (**Figures 3E, G**). To test whether the BPV E5 oncoprotein could interact with STING, we performed co-immunoprecipitation studies using an anti-STING antibody. These studies revealed that the BPV E5 oncoprotein interacted with STING (**Figure 2B**), suggesting that the E5 oncoprotein could play a role in negatively regulating STING expression.

### Upstream effectors of the STING pathway: Interferon-inducible protein 16 (IFI16)

**Figure 4:**
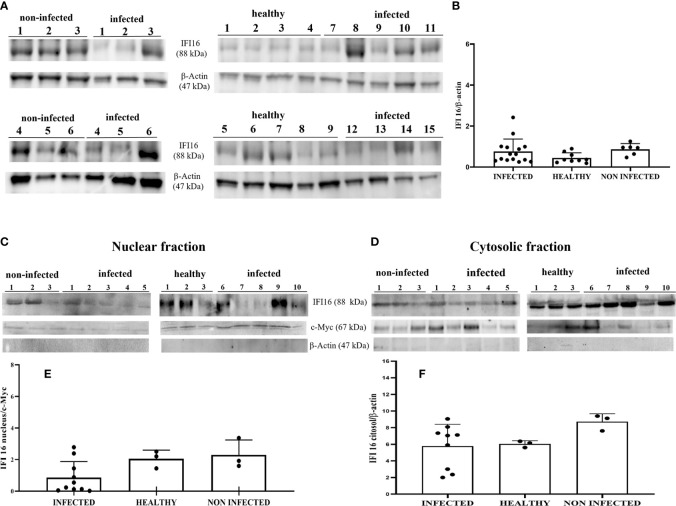
**(A)** Western blot analysis of IFI16 in infected, non-infected, and healthy bladder tissues. **(B)** Densitometric analysis was performed by comparing the protein expression levels of total IFI16 with β-actin. **(C, D)** IFI16 western blot of nuclear and cytosolic fractions of infected, non-infected, and healthy bladder tissues. **(E, F)** cMyc and β-actin were used to verify selectivity of the extracts and perform the densitometric analysis. There was no statistically significant variation in IFI16 protein levels both in total and differentiated fractions in diseased bladder tissue compared with both non-infected and healthy bladder samples. Each plot represents the median of three experiment for each sample.

**Figure 5:**
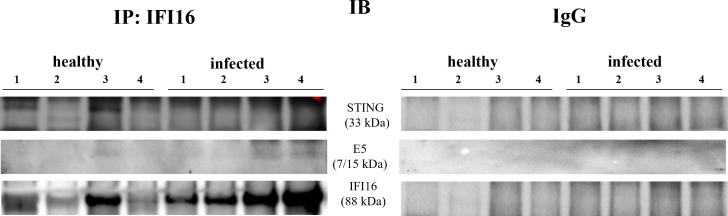
IFI16 immunoprecipitation assay. IFI16 interacted with STING and E5 oncoprotein. The corresponding immunoprecipitation using rabbit IgG is shown at right.

IFI16 is an important innate immune sensor for intracellular DNA; however, the link between nuclear IFI16 and STING remains unknown (36). Since IFI16, localized mainly to the nucleus, was also distinctly detected in the cytoplasm upon DNA virus infection (12, 37), we investigated its expression levels in total and fractionated cellular extracts. WB analysis failed to detect any significant differences in IFI16 expression levels in both total extracts and subcellular fractions (**Figures 4A–F**). It is worth noting that IFI16 expression appears to correlate with papillomavirus transcription and replication (38, 39). Co-immunoprecipitation studies using an anti-IFI16 antibody revealed a ternary complex composed of STING and BPV E5 oncoprotein. Interaction of IFI16 with STING was clearly observed in BPV-negative urothelial cells, whereas it was barely detectable in BPV-infected urothelial cells (**Figure 5**).

### Downstream effectors of the STING pathway

Upon conformational changes resulting in its activation, STING translocates from the ER to the ER-Golgi intermediate compartments (ERGIC), a system composed of tubular structures and vesicles (40). To date, the direct role of STING-induced type I IFNs in antiviral responses has not yet been studied *in vivo* (41). Furthermore, how STING interacts with NF-κB pathway components at the molecular level remains poorly understood (2).

**Figure 6:**
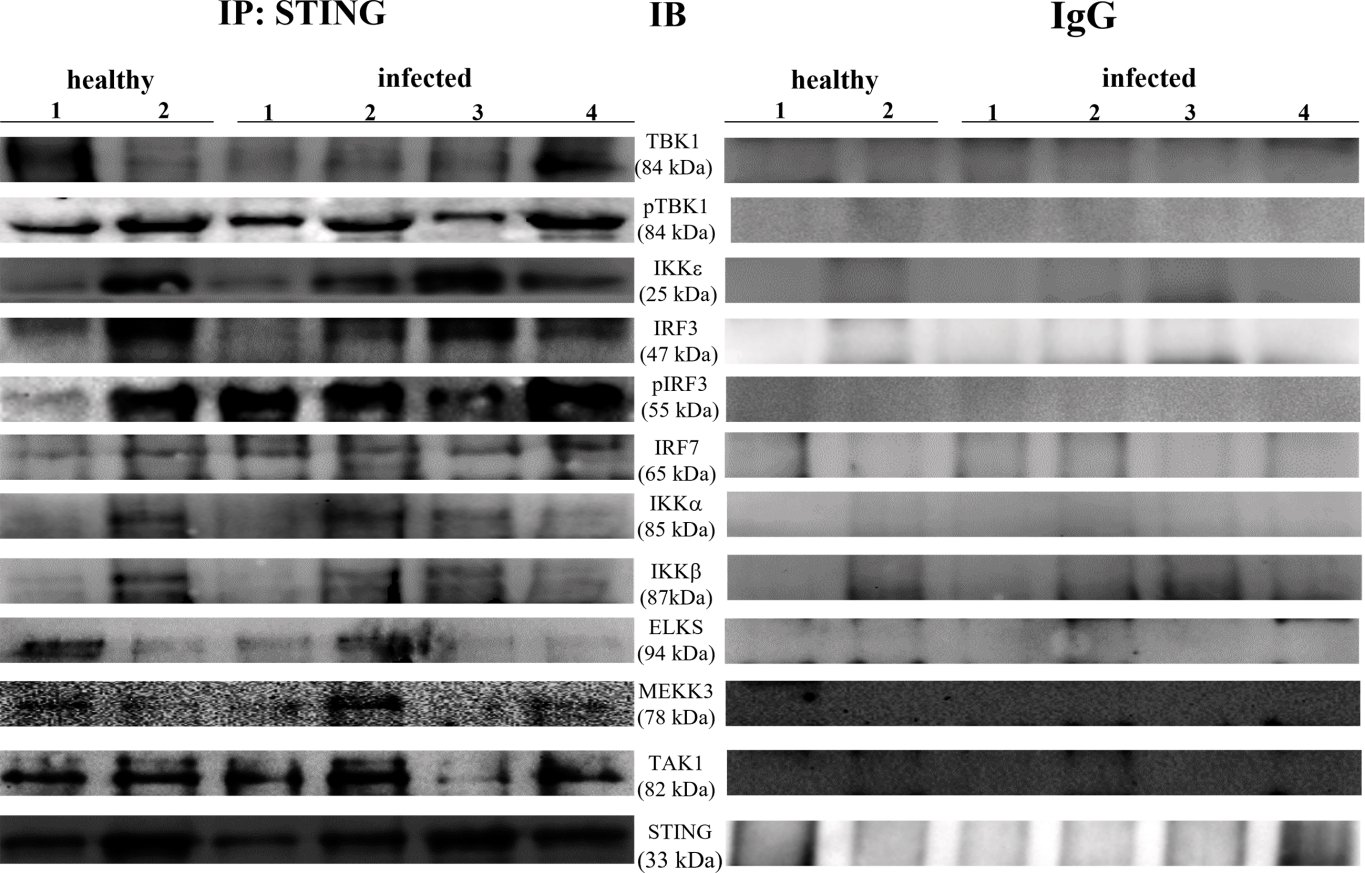
STING immunoprecipitation assay. STING interacted with total and phosphorylated-TBK1 (pTBK1), IKKε, total and phosphorylated-IRF3 (pIRF3), IRF7, IKKα, IKK β, ELKS, MEKK3, and TAK1. The corresponding immunoprecipitation assay using rabbit IgG is depicted in the right.

**Figure 7:**
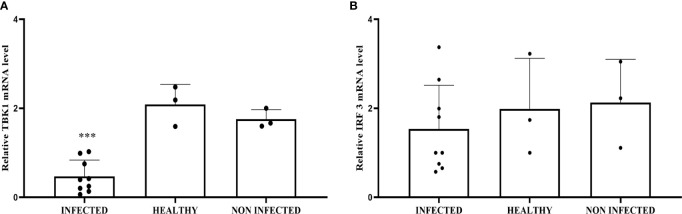
**(A)** RT-qPCR analysis of *TBK1* mRNA levels in infected, non-infected, and healthy bladder samples, respectively. *TBK1* mRNA expression was significantly reduced in infected samples compared with healthy bladder (***p ≤ 0.001). **(B)** RT-qPCR analysis of *IRF3* mRNA levels in infected, non-infected, and healthy bladder samples, respectively. There was no statistically significant variation in *IRF3* mRNA expression level in infected bladder tissues compared with that in both non-infected and healthy bladder samples.

**Figure 8:**
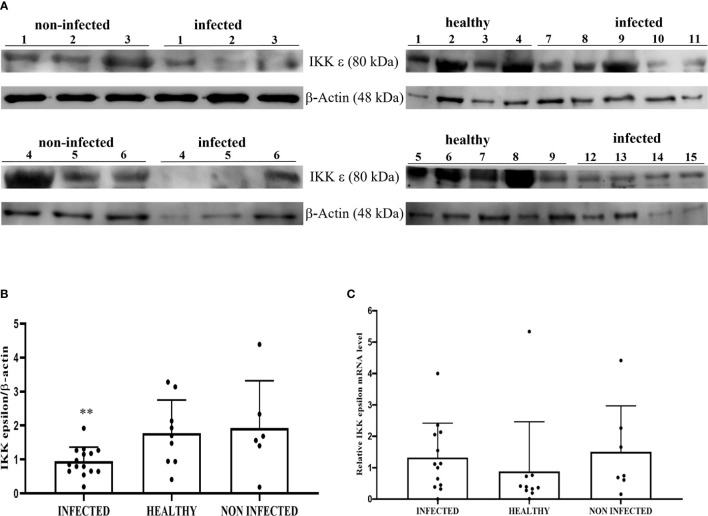
**(A)** Western blot analysis of total IKKε protein in infected, non-infected, and healthy bladder samples and **(B)** Densitometric analysis of IKKε protein relative to β-actin protein level (**p ≤ 0.01). Plots present values found for each sample performed in triplicate. **(C)** RT-qPCR analysis of *IKK*ε mRNA levels in infected, non-infected, and healthy bladder samples, respectively. There was no statistically significant variation in *IKK*ε mRNA expression level in diseased bladder tissue compared with both non-infected and healthy bladder samples.

**Figure 9:**
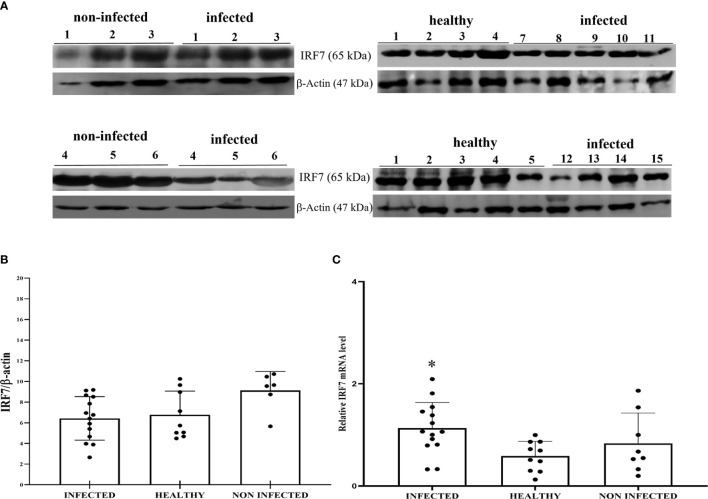
**(A)** Western blot analysis of total IRF7 protein in infected, non-infected, and healthy bladder samples. **(B)** Densitometric analysis of IRF7 protein abundance relative to β-actin protein levels. Plots present values found for each sample performed in triplicate. **(C)** RT-qPCR analysis of *IRF7* mRNA levels in infected, non-infected, and healthy bladder samples, respectively. A significant variation in *IRF7* mRNA expression levels was observed in diseased bladder tissue compared with both non-infected and healthy bladder samples (*p ≤ 0.05).

Co-immunoprecipitation studies using an anti-STING antibody revealed the presence of TBK1, pTBK1, IKKϵ, IRF3, pIRF3, IRF7, IKKα, and IKKβ (**Figure 6**). TBK1 and IKKϵ are members of the IKK family. These kinases are considered master regulators of inflammation and innate immunity *via* control of the transcription factors IRF3, IRF7 and NF-κB during viral infection (42–44). Therefore, we investigated TBK1 mRNA levels using RT-PCR. The sequencing of RT-PCR-generated transcript amplicons (**Supplemental Figure 3A**) revealed cDNA fragments showing 100% identity with the *Bos taurus* TBK1 mRNA sequence deposited in GenBank (Accession number: NM_001192755.1) (**Supplemental Figure 3C**). RT-qPCR revealed a significant reduction in TBK1 mRNA levels in BPV-infected cells compared with that in BPV-uninfected and healthy urothelial cells (***p ≤ 0.001) (**Figure 7A**), which may be the reason for the significant reduction in the protein expression in the same samples that we already documented. Sequencing of RT-PCR-generated transcript amplicons also revealed cDNA fragments showing 100% identity with *Bos taurus* IKKϵ mRNA sequence deposited in GenBank (Accession number: NM_001075281.2) (**Supplemental Figure 3B**). Furthermore, we investigated IKKϵ expression levels using RT-qPCR and WB analysis. No variation was detected in IKKϵ transcripts between BPV-positive and BPV-negative urothelial cells (**Figure 8C**). WB analysis revealed a significant reduction in IKKϵ expression in BPV-positive neoplastic urothelial cells compared with that in BPV-negative urothelial cells (**p ≤ 0.01) (**Figures 8A, B**). IRF3 is a downstream effector of TBK1 and IKKϵ. Using specific primers for bovine *IRF3*, we performed RT-PCR. Sequencing of the RT-PCR-generated transcript amplicons revealed cDNA fragments showing 100% identity with *Bos taurus* IRF3 mRNA sequence deposited in GenBank (Accession number: NM_001029845.3) (**Supplemental Figures 4A, B**). RT-qPCR showed no significant reduction in IRF3 transcripts in any of the examined samples (**Figure 7B**). Therefore, these findings suggest that the significant reduction in the expression levels of IRF3 protein in BPV-positive neoplastic cells compared with that in BPV-negative and healthy urothelial cells, that we previously reported, may be due to post-translational modifications. IRF7 can be activated by STING and its upregulation correlates with type-1 IFN responses (45). We therefore generated its transcript amplicons by RT-PCR. These sequences showed 100% identity with the *Bos taurus* IRF7 mRNA sequence deposited in GenBank (Accession number: NM_001105040.1) (**Supplemental Figures 4A, C**). IRF7 expression studies by WB did not reveal a significant variation between infected, non-infected, and healthy cells (**Figures 9A, B**). However, RT-qPCR showed a significant increase in IRF7 transcripts in BPV-positive neoplastic urothelial cells (*p ≤ 0.05) **Figure 9C**). We previously reported a significant reduction in *IFN-1* mRNA levels in urothelial cells infected by BPVs when compared to the levels in uninfected urothelial cells of healthy cattle. Therefore, we found no correlation between IRF7 expression and type-1 IFN production. This lack of correlation could be based on the specific cell type. It is worth noting that IRF7 acts in a cell type specific manner (46).

The canonical IkB kinase (IKK) complex is composed of catalytic subunits IKKα and IKKβ, and a regulatory subunit IKKγ (also called NEMO or NF-kB essential modulatory) (47).

**Figure 10:**
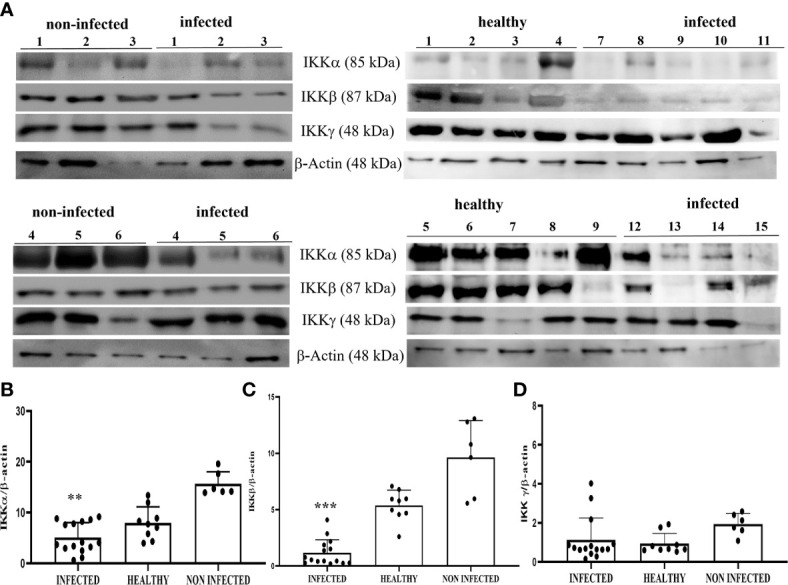
**(A)** Western blot analysis of IKKα, IKKβ, and IKKγ proteins in infected, non-infected, and healthy bladder samples. **(B, C)** Densitometric analysis of IKKα and IKKβ protein levels relative to β-actin protein levels showed a statistically significant difference (**p ≤ 0.01 and ***p ≤ 0.001, respectively) of infected compared with both non-infected and healthy bladder tissue samples. **(D)** Densitometric analysis of IKKγ protein relative to β-actin protein levels: no significant variation was observed. Plots present values found in each sample performed in triplicate.

WB revealed a significant reduction in the expression levels of IKKα and IKKβ in BPV-positive urothelial cells comparison with that in non-infected and healthy cells; no variation was observed in IKKγ expression levels (**p ≤ 0.01; ***p ≤ 0.001) (**Figures 10A–D**). The reduced expression of these proteins was caused at the transcriptional level, as previous RT-qPCR studies of their conjugate mRNAs revealed a significant reduction of transcripts of IKKα, IKKβ, and IKKγ in the same samples. Reduced expression levels of the IKK kinase complex could be a key mechanism of papillomavirus-induced downregulation of NF-κB signaling.

**Figure 11:**
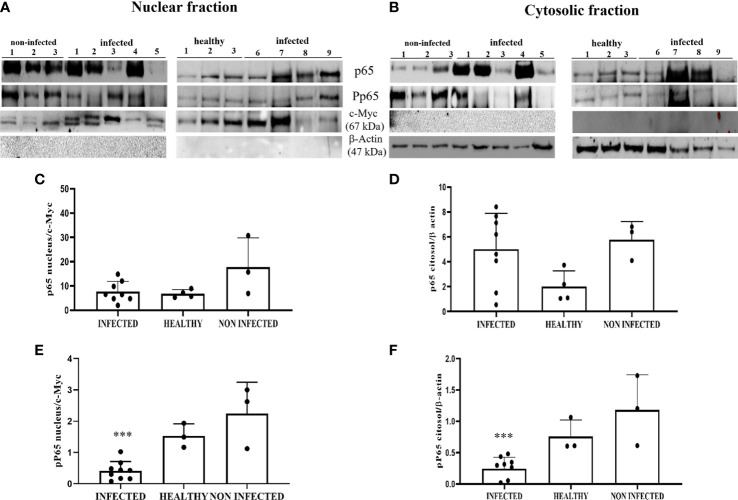
**(A, B)** Nuclear and cytosolic fractions of infected, non-infected, and healthy bladder tissues. The Figure shows western blots of the different fractions of total and phosphorylated p65 (p-p65) proteins. cMyc and β-actin were used to verify selectivity of the extracts and perform densitometric analysis. **(C, D)** Densitometric analysis of the nuclear and cytosolic fractions of total p65 and **(E, F)** phosphorylated p65 (p-p65). Significant differences between BPV-positive and non-infected, healthy nuclear and cytosolic extracts were found for p-p65 (***p ≤ 0.001). Each plot represents the median of three experiments for each sample.

To evaluate NF-κB activation, we investigated the levels of total and phosphorylated p65 (p-p65), a key component of the canonical NF-κB pathway. WB analysis of total protein extracts did not reveal any statistically significant differences between BPV-infected, non-infected, and healthy urothelial cells (data not shown). Next, we carried out an immunoblotting analysis of p65 expression levels in the nuclear and cytosolic fractions. No significant differences were observed in either fraction, even if p65 appeared to be slightly decreased in infected cells compared with that in uninfected urothelial cells (**Figures 11A–D**). WB revealed a significant reduction in p-p65 in both the nuclear and cytosolic compartments of BPV-infected cells compared with that of non-infected and healthy urothelial cells (***p ≤ 0.001) (**Figures 11A, B, E, F**). The lack of activation of the IKK complex could be responsible for reduction in phosphorylated p65, as it has been shown that IKK-mediated p65 phosphorylation at Ser536 is critical for NF-κB-dependent gene expression (48).

**Figure 12:**
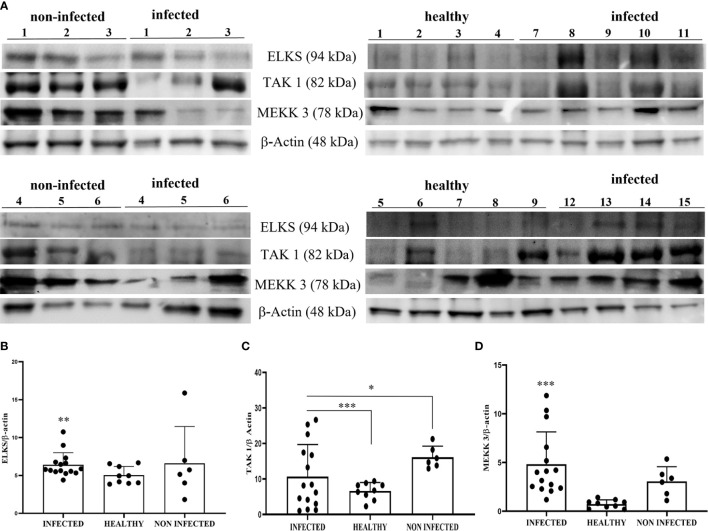
**(A)** Western blot analysis of ELKS, TAK1, and MEKK3 proteins in infected, non-infected, and healthy bladder samples. Densitometric analysis of ELKS **(B)**, TAK1 **(C)**, and MEKK3 **(D)** protein levels relative to β-actin protein levels. Each protein exhibited a significant increase in infected bladder samples compared with both non-infected and healthy samples (*p ≤ 0.05; **p ≤ 0.01; ***p ≤ 0.001). Plots present values found for each sample performed in triplicate.

Recently, it has been suggested that the ELKS protein regulates intracellular transport at the Golgi complex level and may provide molecular scaffolding for organizing vesicle traffic (47, 49, 50). Furthermore, ELKS, an IκB kinase regulatory subunit, is important for the function of the downstream pathway of STING resulting in activation of canonical NF-κB signaling (51–53). ELKS expression correlates with the regulatory function of IKK activation (51). Therefore, we investigated the involvement of ELKS in the STING pathway in BPV infection. First, we speculated that ELKS could be a partner of STING. Co-immunoprecipitation studies using an anti-STING antibody revealed the presence of ELKS (**Figure 6**). Next, we studied ELKS expression levels by WB, which revealed a significant overexpression of this protein in BPV-infected urothelial cells compared with that in healthy urothelial cells (** p ≤ 0.01)) (**Figures 12A, B**). As ELKS expression did not appear to be associated with IKK activation in this study, our data may suggest a novel function of ELKS in STING trafficking. TAK1 and MEKK3 are putative kinases required for STING-mediated NF-κB responses (52, 54). They are upstream kinases responsible for IKKα and IKKβ activation. When overexpressed in mammalian cells, TAK1 and MEKK3 activate IKKs (44, 55). We also detected TAK1 and MEKK3 in the anti-STING immunoprecipitate (**Figure 6**). Therefore, we investigated the expression levels of TAK1 and MEKK3 proteins using WB and found that they were significantly overexpressed in BPV-positive urothelial cells than in uninfected and healthy urothelial cells (^*^p ≤ 0.05; *** p ≤ 0.001) (**Figures 12A, C, D**). As in our study, we did not identify any IKK activation, which is in complete agreement with previous studies (52), suggesting that the involvement of TAK1 and/or MEKK3 in IKK activation may be cell type-specific.

**Figure 13:**
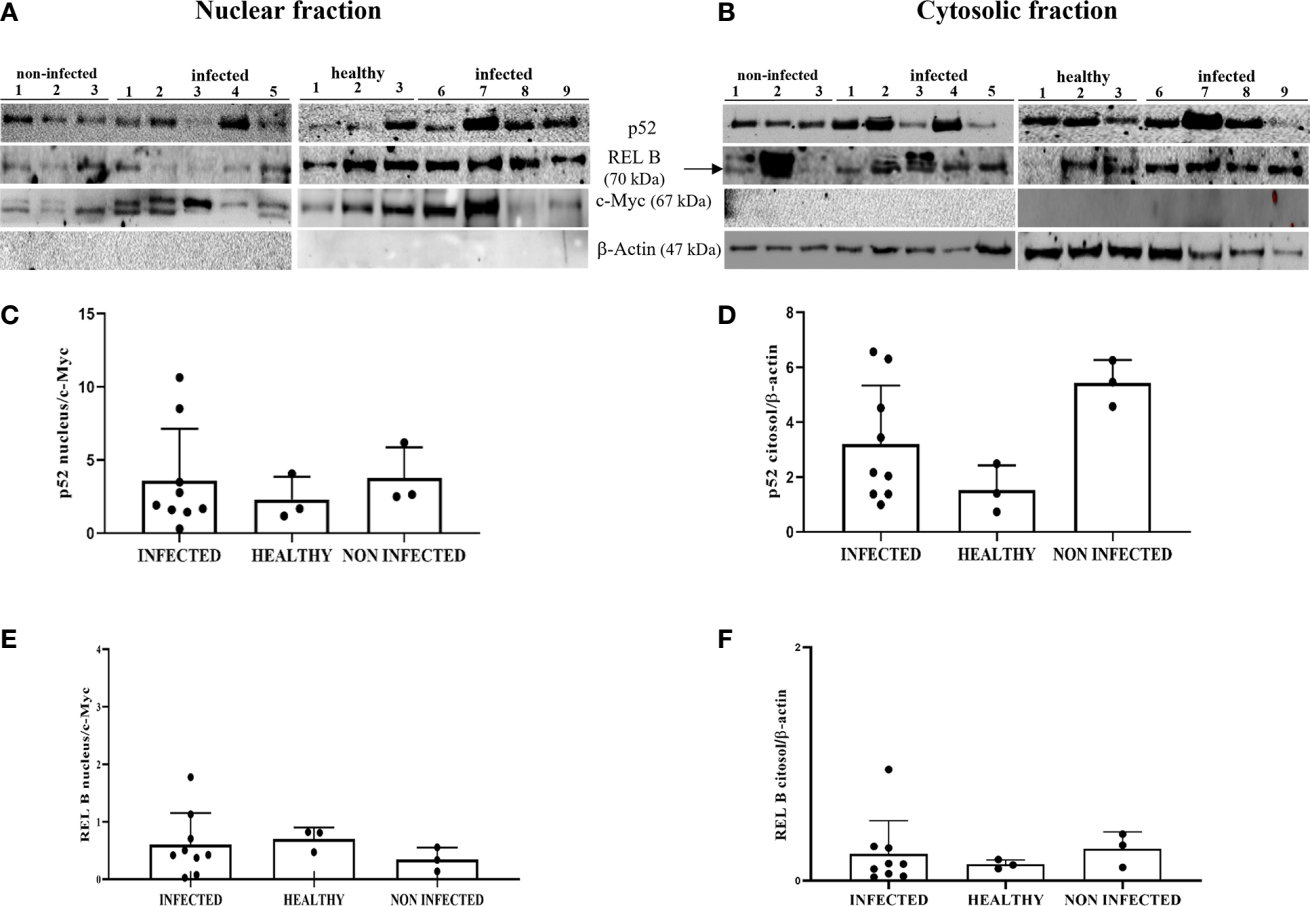
**(A, B)** Nuclear and cytosolic fractions of infected, non-infected, and healthy bladder tissues. The Figure presents western blots of the different fractions of the proteins p52 and RelB. cMyc and β−actin were used to verify the selectivity of the extracts and perform densitometric analysis. **(C, D)** Densitometric analysis of the nuclear and cytosolic fractions of p52 and **(E, F)** RelB. No significant variation is seen in the p52 and RelB protein levels in diseased bladder tissues compared with both non-infected and healthy bladder samples. Plots present values found for each sample performed in triplicate.

Non-canonical NF-κB can be activated in the cGAS-STING axis by triggering p52 nuclear translocation, thus limiting type I IFNs and canonical NK-κB (56, 57). We analyzed the expression levels of p52 and RelB subunits, an essential p52 binding partner, in both the nuclear and cytosolic fractions by WB. This investigation did not detect any significant changes in the expression levels of these proteins in the subcellular fractions of BPV-infected cells compared with that in uninfected urothelial cells (**Figures 13A–F**). It is plausible that non-canonical NF-κB is not involved in the STING pathway in this spontaneous model of papillomavirus disease in cattle; however, we do not exclude the possibility that it further contributes to negatively regulating STING effector mechanisms, which are already impaired in BPV-positive urothelial cells.

## Discussion

This study provides novel mechanistic insights into the potential role of E5 oncoprotein in dysregulating host antiviral innate immune responses in a spontaneous model of BPV infection. Persistent infection and tumorigenesis by PVs are known to require viral manipulation of a variety of cellular processes, including those involved in innate immune responses. Induction of immune evasion by BPV is one of the most important mechanisms in persistent BPV infection and is responsible for BPV-associated disease progression. This study describes a redundant strategy employed by BPV to thwart cGAS-STING-mediated host innate immune responses, leading to abnormal cellular antiviral defence, in a spontaneous model of viral disease in cattle. Our findings revealed that the BPV E5 oncoprotein interacts with STING, and forms a ternary complex along with IFI16. It is also conceivable that the E5 oncoprotein acts at the transcriptional level, reducing cytoplasmic STING expression in this spontaneous model of BPV disease. Our *in vivo* findings are in line with experimental *in vitro* studies showing that the HPV18 E6 and E7 oncoproteins are responsible for STING transcriptional reduction (31). Furthermore, BPV E5 may dysregulate organelle acidification thus being responsible for an adverse cellular environment that interferes with inter-organelle STING trafficking and function. Organelle acidification, such as acidified endolysosomes, is a biochemical feature essential for STING trafficking that leads to its activation (33). The BPV E5 oncoprotein can bind to subunit D of the V_1_-ATPase proton pump, thus impairing its activity and dysregulating lysosomal acidification (58). Imbalances in intracellular trafficking routes may have profound effects on STING activity (57). Furthermore, aberrant trafficking of STING could explain why its canonical function and subsequent downstream signaling are compromised during viral infection (59).

We showed that the cGAS-STING signaling pathway was impaired and RLRs were downregulated in PV-infected cells. Therefore, cGAS-STING and RLRs could act in a redundant and/or synergistic manner to mediate innate immune responses against BPV infection. Recently, crosstalk between RNA viruses and DNA sensors has been shown, which is improving our understanding of the interconnectedness of the cGAS-STING pathway with RLR signaling (60, 61).

BPVs reduce the levels of DNA sensors that can recognize them, which can hamper pTBK1 and IKKϵ signaling, as well as the production of IFNs, similar to human PVs (62). IFN production plays a crucial role in the immune response against PV infection, as IFNs promote the clearance of latent PV episomes in persistently infected cells and/or promote a rapid reduction in PV episome copies per cell. Reportedly, basal cells in the initial infection usually contain low levels (approximately 100 copies per cell) of human and bovine PV episomes (63, 64). Animal cells that fail to resolve their infection and retain oncogene expression for years can facilitate tumorigenesis *via* BPVs.

Furthermore, this study showed that the canonical IKK complex was downregulated at the transcriptional level in BPV-positive neoplastic urothelial cells. IKK proteins are key components required for NF-κB activation, which suggests that the BPV E5 oncoprotein could negatively influence NF-κB activation. In the current study, we observed a statistically significant reduction in phosphorylated p65, the nuclear localization of which is a principal control point for NF-κB-induced gene expression, thus providing molecular responses to pathogens or inflammatory stimuli (65). Our findings corroborated experimental data showing that HPV oncoproteins are able to downregulate NF-κB activation, thus contributing to viral escape from the immune system (51). However, the molecular mechanisms leading to transcription factor induction are not completely understood and this remains an important area for the future as relatively little progress has been made to understand how STING signaling delineates the molecular events that follow it (2, 4).

In conclusion, a better understanding of the mechanisms of early viral clearance and the development of approaches to induce viral clearance are important for the prevention of persistent infections and subsequent variable manifestations of disease by BPVs.

Finally, it is considered that many aspects of PV biology can only be fully understood using animal models (70). The BPV model has been useful in assigning key functions to many of the early (E) proteins, despite the fact that delta BPVs, unlike HPVs, replicate in and transform both fibroblasts and epithelial cells (66). When used as part of a multi-disciplinary approach, this spontaneous model of PV disease will continue to play a significant role in developing our understanding of virus biology.

## Data availability statement

The datasets presented in this study can be found in online repositories. The names of the repository/repositories and accession number(s) can be found in the article/**Supplementary Material**.

## Ethics statement

In this study, animal experiments were not performed. All the samples were collected post-mortem from slaughterhouses, and therefore, no ethical approval was required.

## Authors contributions

SR designed the experiments, AC, FDF, BC, and BDU carried out the experiments, SR and AFC analyzed data, SR wrote the manuscript. All authors read and approved the final manuscript.

## Funding

This research was partially supported by Regione Basilicata and Istituto Zooprofilattico Sperimentale del Mezzogiorno. The funders of the work did not influence study design, data collection and analysis, decision to publish, or preparation of the manuscript.

## Acknowledgments

The authors wish to thank Dr G. Salvatore of the Regione Basilicata, Dr S. Morace of the University of Catanzaro ‘Magna Graecia’, Drs F. Di Domenico, E. Grieco, G. Milone, F. Di Santi from Azienda Sanitaria Locale (ASL) of Salerno, Dr R.N. La Rizza from ASL of Vibo Valentia, and Dr Marcellino Riccitelli for their technical help.

## Conflict of interest

The authors declare that the research was conducted in the absence of any commercial or financial relationships that could be construed as a potential conflict of interest.

## Publisher’s note

All claims expressed in this article are solely those of the authors and do not necessarily represent those of their affiliated organizations, or those of the publisher, the editors and the reviewers. Any product that may be evaluated in this article, or claim that may be made by its manufacturer, is not guaranteed or endorsed by the publisher.
