# Supplementary material for: Bovine delta papillomavirus E5 oncoprotein negatively regulates the cGAS-STING signaling pathway in cattle in a spontaneous model of viral disease
Source: Front Immunol. 2022 Oct 12;13:937736. doi: 10.3389/fimmu.2022.937736 (PMC9597257; doi:10.3389/fimmu.2022.937736)
Supplement: Supplementary file 1 [file DataSheet_1.zip › legends of supplementary figures.DOCX]

**Supplemental Figure 1** (A): One-step reverse transcription (RT)-ddPCR. No E5 mRNAs related to four bovine Delta papillomavirus were detected using one-step RT-ddPCR in six “non-infected” and (B) nine healthy bladder samples of cattle. (A): Channel G04: positive control characterized by presence of blue droplets; H04: negative control characterized by grey droplets; (B): Channel G03: positive control characterized by presence of blue droplets; H03: negative control characterized by grey droplets.

**Supplemental Figure 2** (A): Electrophoresis of RT-PCR products for evaluating cGAS and STING mRNA expression in healthy and infected tissues. MW: DNA molecular weight marker (100 bp ladder); C-: RT-PCR negative control (B): Transcript amplicons showed 100% identity with *Bos taurus* cGAS transcript variant X1 sequence deposited in GenBank (Accession number: XM_002690020.6). (C) Transcript amplicons showed 100% identity with *Bos taurus* STING1 mRNA sequence deposited in GenBank (Accession number: NM_001046357.2).

**Supplemental Figure 3** (A) Electrophoresis of RT-PCR products to evaluate *IKK*ε and *TBK1* mRNA expression in healthy and infected tissues. MW: DNA molecular weight marker (100 bp ladder). C-: RT-PCR negative control. (B) Transcript amplicons showed 100% identity with *Bos taurus IKKε* mRNA sequence deposited in GenBank (Accession number: NM_001075281.2). (C) Transcript amplicons showed 100% identity with *Bos taurus* *TBK1* mRNA sequence deposited in GenBank (Accession number: NM_001192755.1).

**Supplemental Figure 4** (A) Electrophoresis of RT-PCR products for evaluating *IRF7* and *IRF3* mRNA expression in healthy and infected tissues. MW: DNA molecular weight marker (100 bp ladder). C-: RT-PCR negative control. (B) Transcript sequences showed 100% identity with *Bos taurus IRF7* mRNA sequence deposited in GenBank (Accession number: NM_001105040.1). (C) Transcript sequences showed 100% identity with *Bos taurus IRF3* mRNA sequence deposited in GenBank (Accession number: NM_001029845.3).
