# Supplementary figures and images for: Bovine delta papillomavirus E5 oncoprotein negatively regulates the cGAS-STING signaling pathway in cattle in a spontaneous model of viral disease
Source: Front Immunol. 2022 Oct 12;13:937736. doi: 10.3389/fimmu.2022.937736 (PMC9597257; doi:10.3389/fimmu.2022.937736)

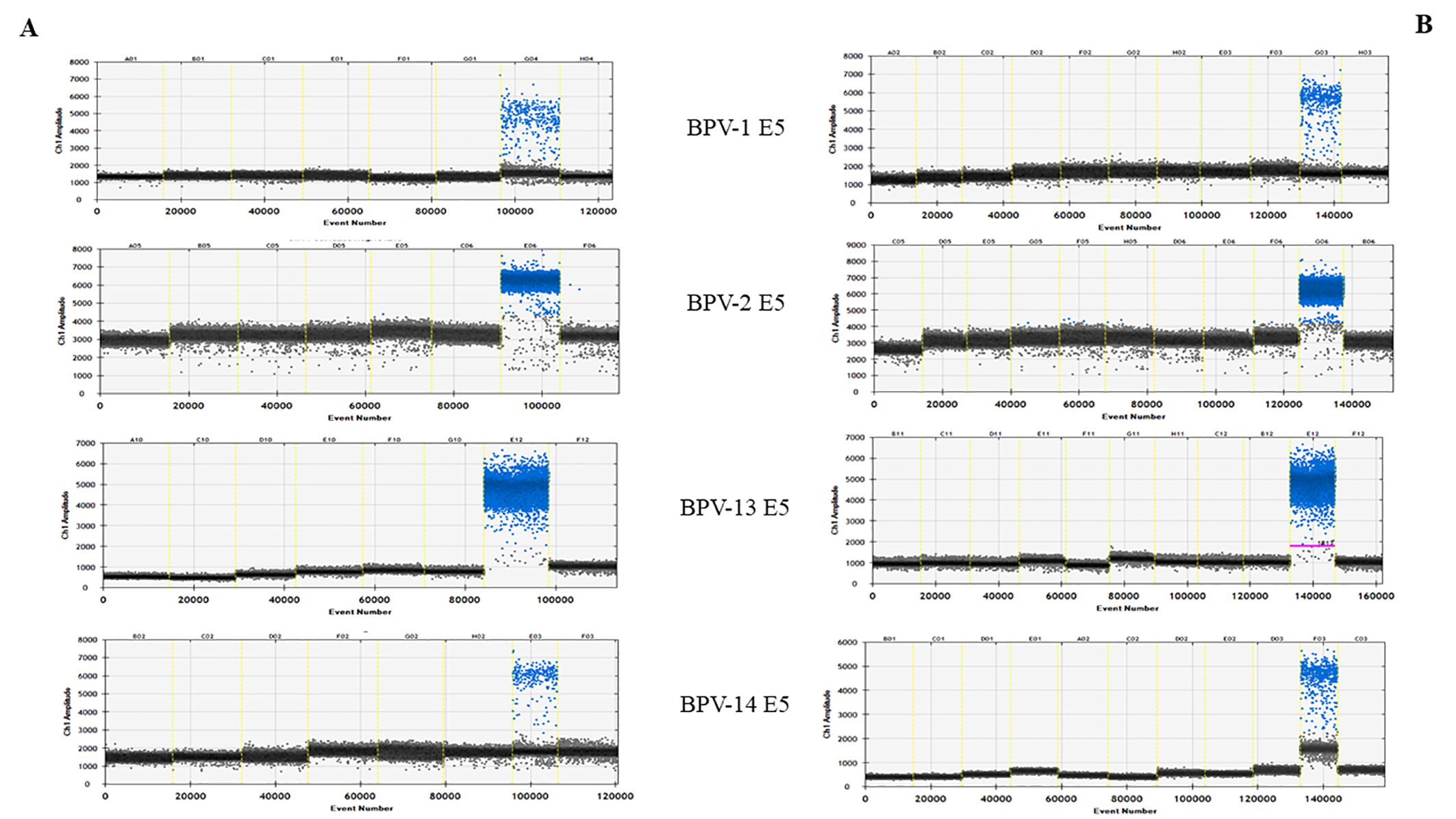

Supplement: Supplementary file 1 [file DataSheet_1.zip › Supplemental Figure 1.TIF]

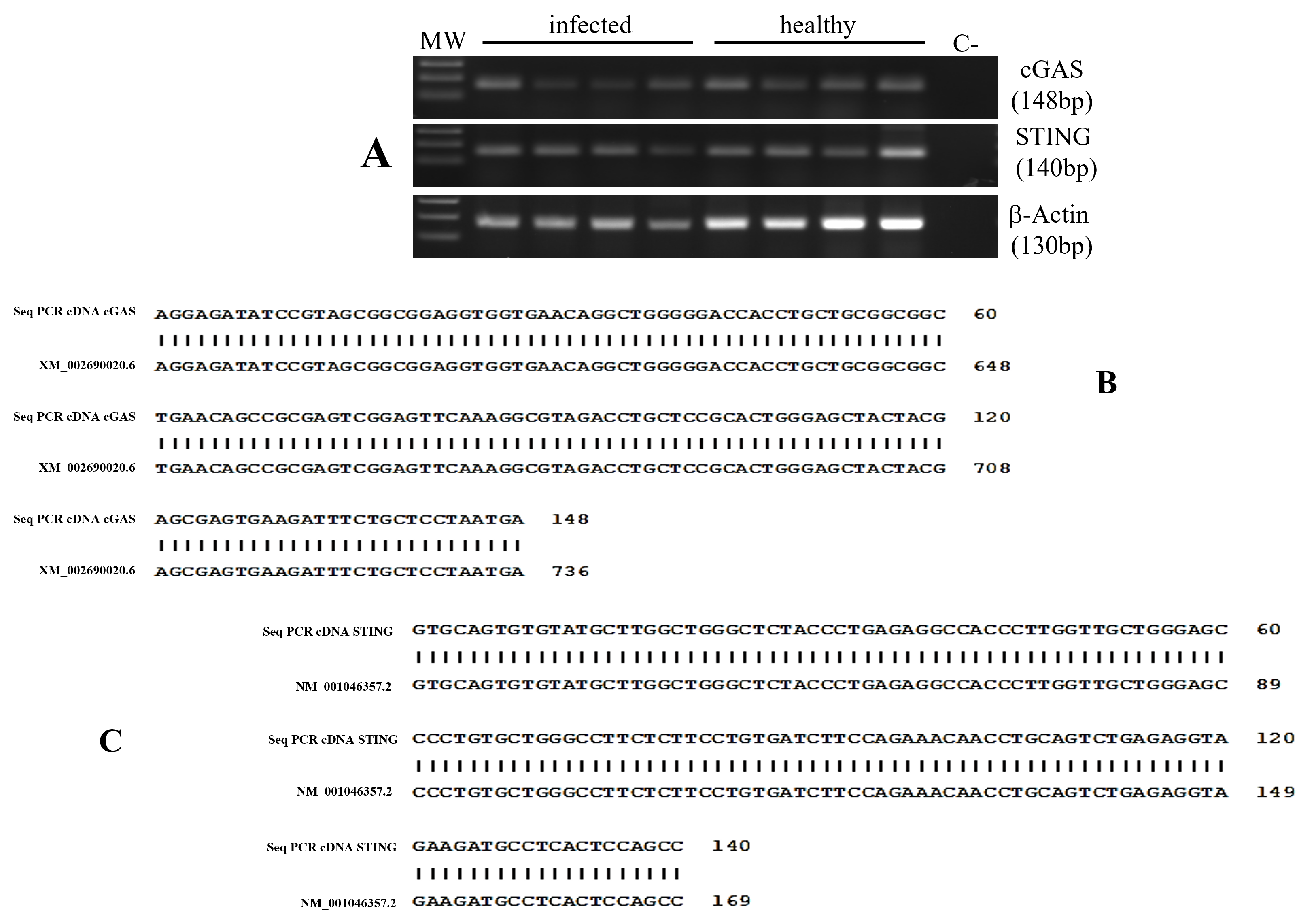

Supplement: Supplementary file 1 [file DataSheet_1.zip › Supplemental Figure 2.tif]

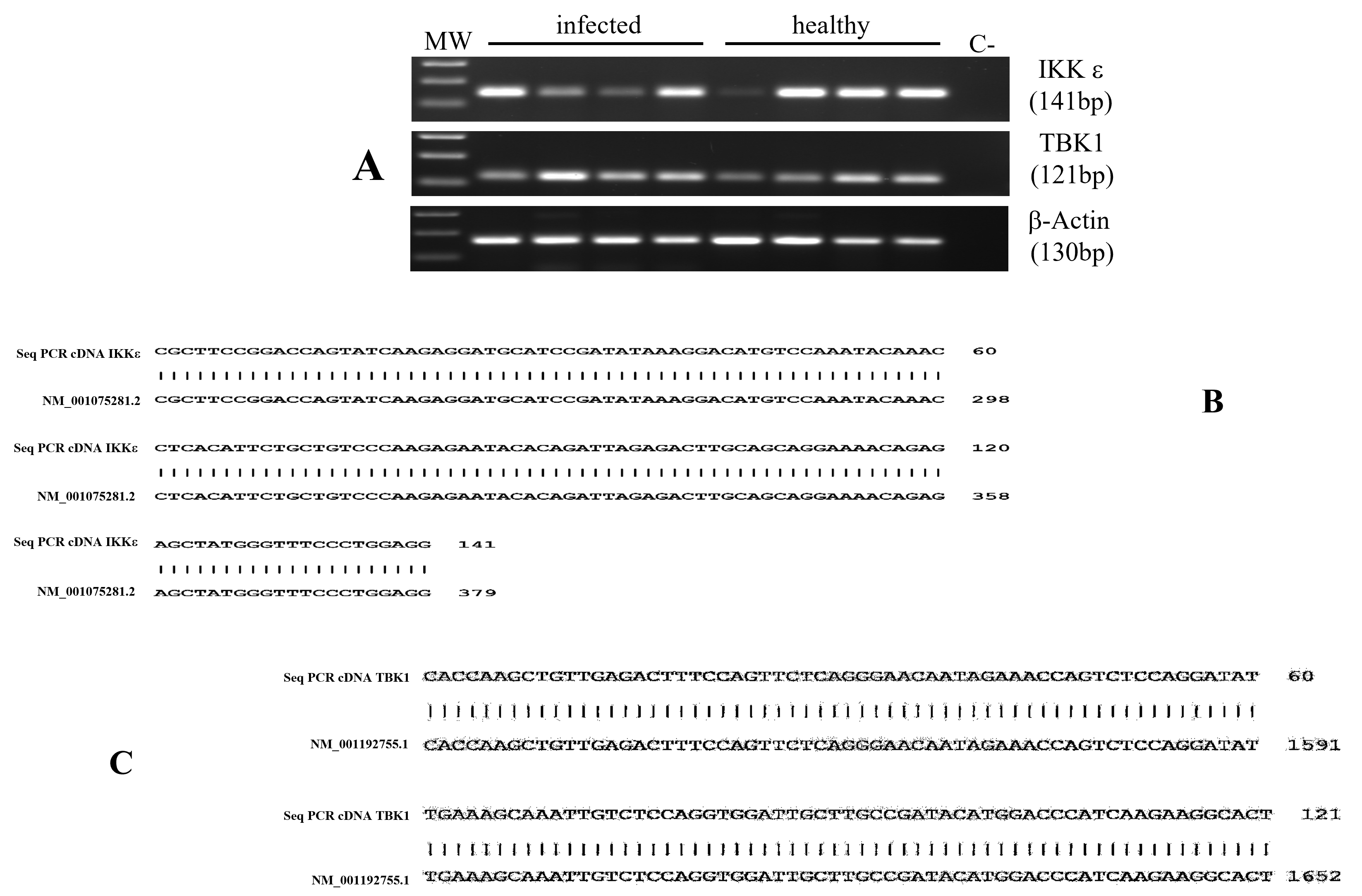

Supplement: Supplementary file 1 [file DataSheet_1.zip › Supplemental Figure 3.tif]

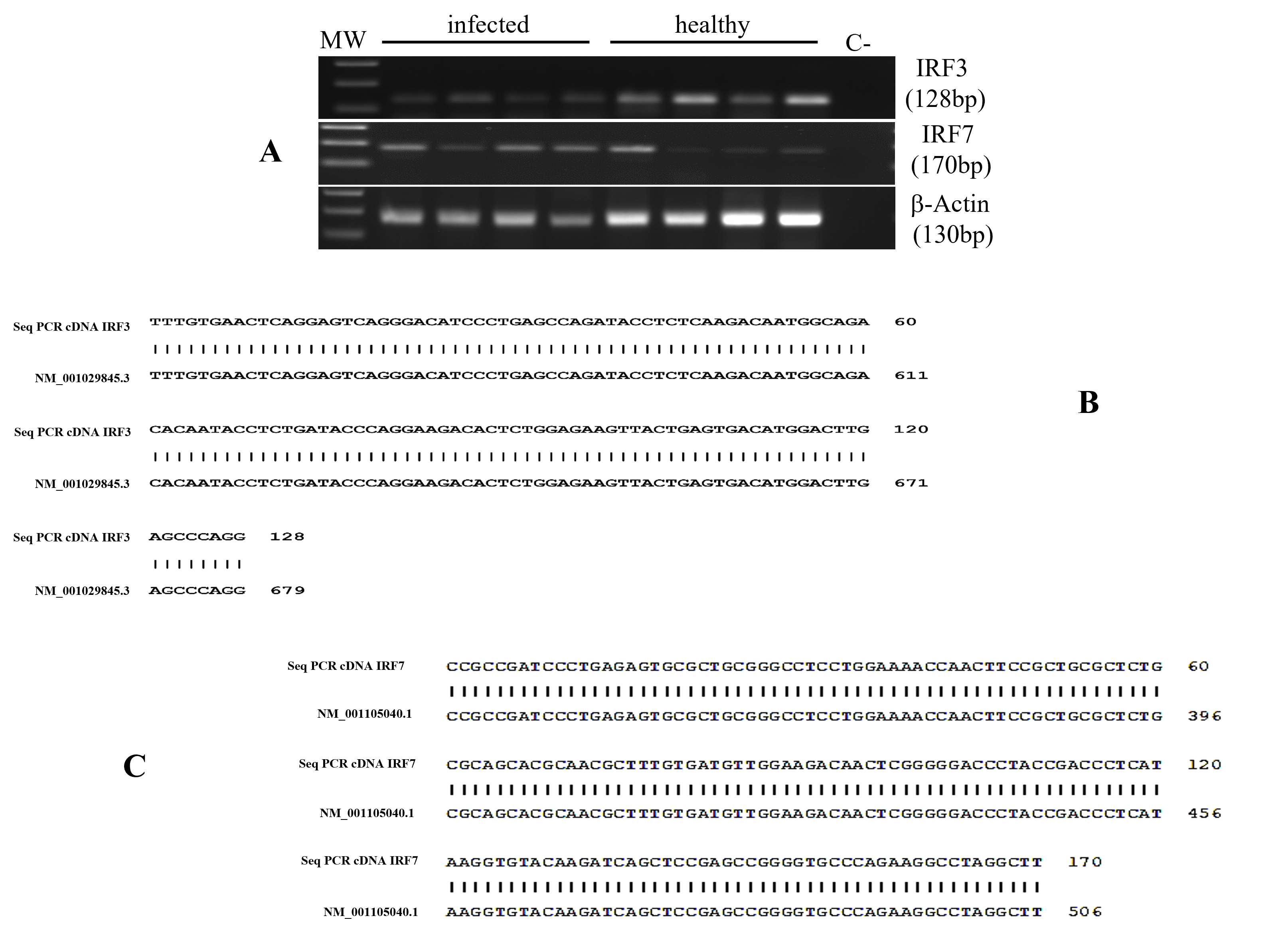

Supplement: Supplementary file 1 [file DataSheet_1.zip › Supplemental Figure 4.tif]
